# Scents in the stack: olfactometric proficiency testing with an emission simulation apparatus

**DOI:** 10.1007/s11356-018-2515-z

**Published:** 2018-06-20

**Authors:** Stephan Stöckel, Jens Cordes, Benno Stoffels, Dominik Wildanger

**Affiliations:** Department I3 (Air Pollution Control, Emission), Hessian Agency for Nature Conservation, Environment and Geology, Kassel, Germany

**Keywords:** Olfactometry, Odor threshold, Proficiency test, Emission simulation apparatus, Stack emission, EN 13725

## Abstract

Olfactometry is globally acknowledged as a technique to determine odor concentrations, which are used to characterize odors for regulatory purposes, e.g., to protect the general public against harmful effects of air pollution. Although the determination procedure for odor concentrations is standardized in some countries, continued research is required to understand uncertainties of odor monitoring and prediction. In this respect, the present paper strives to provide answers of paramount importance in olfactometry. To do so, a wealth of measurement data originating from six large-scale olfactometric stack emission proficiency tests conducted from 2015 to 2017 was retrospectively analyzed. The tests were hosted at a unique emission simulation apparatus—a replica of an industry chimney with 23 m in height—so that for the first time, conventional proficiency testing (no sampling) with real measurements (no reference concentrations) was combined. Surprisingly, highly variable recovery rates of the odorants were observed—no matter, which of the very different odorants was analyzed. Extended measurement uncertainties with roughly 30–300% up to 20–520% around a single olfactometric measurement value were calculated, which are way beyond the 95% confidence interval given by the widely used standard EN 13725 (45–220%) for assessment and control of odor emissions. Also, no evidence has been found that mixtures of odorants could be determined more precisely than single-component odorants. This is an important argument in the intensely discussed topic, whether *n*-butanol as current reference substance in olfactometry should be replaced by multi-component odorants. However, based on our data, resorting to an alternative reference substance will not solve the inherent problem of high uncertainty levels in dynamic olfactometry. Finally, robust statistics allowed to calculate reliable odor thresholds, which are an important prerequisite to convert mass concentrations to odor concentrations and vice versa.

## Introduction

The emission of malodors into the environment has numerous adverse effects like annoyance, health effects, or depreciation of property values. The public awareness to environmental odors emitted from agricultural, municipal, or industrial sources has therefore increased over the last decade. As a consequence, odor is classified as atmospheric pollutant by many jurisdictions and odor measurement and regulation is handled by local jurisdictions, states, provinces, and countries in different manners (Brancher et al. 2017).

In odor-regulating countries, odor evaluation is usually performed by collecting air samples at the source of emission, measuring odor concentration by means of dynamic olfactometry (olfaction—the sense of smell) or the triangle bag method in Japan, and finally predicting odor concentrations at nearby receptors employing atmospheric dispersion models. Although dynamic olfactometry is standardized, e.g., in the USA (ASTM E679-04 2010), in Europe (EN 13725 2003), and in Australia and New Zealand (AS/NZS 4323.3 2001), continued research is required to understand uncertainties with odor monitoring and prediction. An intrinsic challenge and important origin of uncertainty is that the measured response is a perception commonly registered by human panelists with individual sensitivities towards odors. In an attempt to reduce this effect, panelists are selected based on their sensitivity to one or more standard chemicals. The European Standard EN 13725 for example uses *n*-butanol as sole reference standard. The Japanese method, however, which is applied on national level through the Offensive Odor Control Law, requires assessors to undertake an aptitude test using five standard odorants (2-phenylethanol, maple lactone, isovaleric acid, *γ*-decalactone, skatole) to ensure that assessors reflect the average odor perception of a “normal” population (Brancher et al. 2017). Whether these criteria are sufficient is still up for debate (Klarenbeek et al. 2014). If olfactometrically measured odor concentrations have to be converted into chemical concentrations, the odor threshold concentration of the odorous substance usually needs to be known, as it is a measure of how effective an airborne chemical can elicit an effect on the olfactory system of a human subject. Unfortunately, referenced threshold values can differ over several orders of magnitude (Abraham et al. 2012; Wu et al. 2017). Other causes for uncertainty might be due to odor sampling, changes to the samples during storage, and dilution in the olfactometer (Hansen et al. 2016; Kasper et al. 2017). Several publications have already addressed these shortfalls. Especially, proficiency testing has played a major role in establishing and validating standards for olfactometry to attain defined levels of statistical performance parameters such as precision under repeatability conditions and accuracy (Maxeiner 2006; Van Harreveld et al. 2009). Most of the publications, however, focus solely on uncertainties caused by the analytical process, such as variabilities among individual panelists, inter-panel variability, within-panel variability, and inter-laboratory variability (Clanton et al. 1999; Hove et al. 2016; McGinley and McGinley 2006). Accuracy is also an important contributor to uncertainty, which in essence cannot be measured for real samples.

This is where the presented work kicks in. It amalgamates conventional proficiency tests (no sampling) with real measurements (no reference concentrations) by using an in-house-constructed emission simulation apparatus (ESA), which is designed as a replica of an industrial chimney (Cordes et al. 2015). In this ESA, gas flows are loaded with defined amounts of odors. Flow parameters and mass concentrations are hereby precisely kept reproducible and constant. This makes the ESA a perfect venue for olfactometric proficiency tests as participants are provided with well-defined gas conditions and concentrations. Consequently, the process of sampling and olfactometric determination of odor concentrations is assessed as a whole and delivers results, which are much more informative and representative than measurements on genuine locations of emissions would be. Due to the strict process control and surveillance, a comparison of results from different proficiency test rounds is feasible, as well as a structured search for possible impact factors on the results.

The first six proficiency tests of this kind have been conducted in 2015, 2016, and 2017. The presented paper reports on the achieved results, where six different odorants—including two mixtures—were analyzed. The shown outcomes bear on a retrospective analysis of the cumulated data of all six proficiency tests regarding the following issues: Are the determined odor thresholds—calculated via a robust consensus algorithm—in line with published values? How large are the measurement uncertainties and the reproducibility variance for each odorant? Right now, EN 13725, which is widely used in Europe for assessment and control of odor emissions, focuses only on quality criteria for single laboratories. Do the obtained results reflect the amount of variability, which had been achieved in previous olfactometric proficiency tests, taking into account that this time, also sampling was part of the process? Is it advisable to hang on to the idea that performance characteristics for certain odors are predictable by looking solely at the performances to the reference *n*-butanol?

## Materials and methods

### General

The data presented in this publication was generated during six proficiency tests (RV429O and RV430O in 2015, RV451O and RV452O in 2016, and RV479O and RV480O in 2017) organized by Dezernat I3 – Luftreinhaltung, Emissionen (Department I3 – Air Pollution Control, Emission), Hessisches Landesamt für Naturschutz, Umwelt und Geologie (HLNUG, Hessian Agency for Nature Conservation, Environment and Geology) in Kassel, Germany. All concentrations relate to olfactometric normal conditions (*p* = 1,013.25 mbar, *T* = 293.15 K). All participants were accredited according to ISO/IEC 17025 and EN 13725, they and their measurement results are presented in anonymized form. All participants carried out the sampling and olfactometry conforming to EN 13725, including all required quality assurance and quality control measures. Twenty-nine of them used transportable odournet TO8 olfactometers, 7 used transportable Ecoma TO7 olfactometers, and 2 participants used other models. The size of the panel used ranged from 4 to 7 panelists, with an average of 4.8 (16 participants used 4, 16 used 5, 4 used 6, and 2 used 7 panelists). Samples were collected in PET-bags and transported in light impenetrable containers or bags. Olfactometry was completed within 6 h after sampling, conforming to guideline VDI 3880 (VDI 3880 2011).

### Emission simulation apparatus

A detailed description of the emission simulation apparatus can be found in a previous publication (Cordes et al. 2015). In short, the emission simulation apparatus (ESA) approximates an industrial factory chimney with a round stainless steel conduit with a diameter of 40 cm and a height of 23 m as its key component. Twenty-eight sampling openings, located on two floors, can be used for sampling and measuring pollutants. A stable test atmosphere with a known composition is generated by feeding filtered and heated ambient air (2000–6000 m^3^/h) into the system, before precisely dispensed pollutants are added in a dosing laboratory. Each fed-in amount of liquids and solids is determined gravimetrically; furthermore, continuous measurements allow surveilling the generated concentrations of added components in a real-time manner. This ensures controllable and constant conditions concerning temperature, gas flow velocity, composition of the flue gas, and the concentration of the gas components throughout the whole proficiency test.

### Dosing of odorants

During the six proficiency tests, six different odorants were dosed, namely amyl acetate (abbreviated as “AAC” throughout the manuscript, 99.9%, Sigma-Aldrich Chemie GmbH, 89555 Steinheim, Germany), a mixture of organic, aromatic solvents (“ETX”, ethyl benzene, 99.9%; toluene, 99.9%; *m*-xylene, 99.8%; *o*-xylene, 98.7%; *p*-xylene, 99.6%; all from Merck Chemicals GmbH, 65824 Schwalbach, Germany, used as a 1:1:1:1:1 (m/m) mixture), *n*-butanol (“NBU” purity 99.8%, Merck Chemicals GmbH, 65824 Schwalbach, Germany), pig odor mixture (“PIG”, Olfasense GmbH, 24118 Kiel, Germany), (*R*)-(+)-limonene (“RLI”, 97%, Alfa Aesar GmbH & Co.KG, 76185 Karlsruhe, Germany), and tetrahydrothiophene (“THT”, 99.7%, Sigma-Aldrich Chemie GmbH, 89555 Steinheim, Germany) diluted in ethanol (99.9%, Merck Chemicals GmbH, 65824 Schwalbach, Germany) 1:100 *V*/*V*.

A calibration gas generator (HOVACAL digital 122, IAS GmbH, 58640 Iserlohn, Germany) was used in combination with a precision balance (LP 1200 S, Sartorius AG, 37075 Göttingen, Germany) to dose the odorants into the ESA. The constancy of the dosing was continuously double-checked using a flame ionization detector (Multi-FID 14, ABB Automation GmbH, 68309 Mannheim, Germany) near the end of the ESA conduit system. The volume flow was adjusted to circa 2900 m^3^/h (normal conditions, dry) and constantly monitored using a gas flow measurement orifice (Blende 65856, Hartmann & Braun Meß- und Regeltechnik/ABB Automation Products GmbH, 63755 Alzenau, Germany) in combination with a measuring transducer (AVA 500, Schoppe & Faeser GmbH/ABB Automation Products GmbH, 63755 Alzenau, Germany).

### Execution and evaluation of the proficiency tests

In the course of each proficiency test, the concentration of four different odorants (here also referred to as “components”) needed to be determined (see Tables 1 and 2) by conforming to the current version of EN 13725 and VDI 3880 (EN 13725 2003; VDI 3880 2011). The participants had to carry out all measurements using their own equipment and all participants had to conduct the samplings simultaneously. For each of the four components, three subsequent measurements were carried out, adding up to 12 measurements. The sampling time was generally 10 min. The olfactometry was conducted in the vicinity of the sampling site in suitable mobile or stationary (e.g., in a hotel) odor rooms and measurement results (expressed as ou_E_/m^3^) had to be submitted to HLNUG on the same day the proficiency test took place.

**Table 1:** Test parameters and results for the components AAC, ETX, and NBU: Mean of assigned concentrations per dosage *X*_*k*_, mean of measured concentrations *X*_*k,p*_, and relative standard deviation *σ*^*r*^_*k,p*_

Test	Number of participants	AAC			ETX			NBU		
		*X*_*k*_ [ou_E_/m^3^]	*X*_*k,p*_ [ou_E_/m^3^]	*σ*^*r*^_*k,p*_ [%]	*X*_*k*_ [ou_E_/m^3^]	*X*_*k,p*_ [ou_E_/m^3^]	*σ*^*r*^_*k,p*_ [%]	*X*_*k*_ [ou_E_/m^3^]	*X*_*k,p*_ [ou_E_/m^3^]	*σ*^*r*^_*k,p*_ [%]
429O	9	1897	2803	53	–	–	–	591	811	50
430O	7	1917	1721	67	–	–	–	600	1014	38
451O	5	–	–	–	787	758	46	1073	1078	24
452O	4	–	–	–	1068	1126	20	464	709	37
479O	6	–	–	–	927	676	37	529	457	37
480O	7	–	–	–	516	890	59	763	830	40

**Table 2:** Test parameters and results for the components PIG, RLI, and THT: Mean of assigned concentrations per dosage *X*_*k*_, mean of measured concentrations *X*_*k,p*_, and relative standard deviation *σ*^*r*^_*k,p*_

Test	Number of participants	PIG			RLI			THT		
		*X*_*k*_ [ou_E_/m^3^]	*X*_*k,p*_ [ou_E_/m^3^]	*σ*^*r*^_*k,p*_ [%]	*X*_*k*_ [ou_E_/m^3^]	*X*_*k,p*_ [ou_E_/m^3^]	*σ*^*r*^_*k,p*_ [%]	*X*_*k*_ [ou_E_/m^3^]	*X*_*k,p*_ [ou_E_/m^3^]	*σ*^*r*^_*k,p*_ [%]
429O	9	–	–	–	1711	2084	52	1055	1553	50
430O	7	–	–	–	1762	2040	48	1006	1258	33
451O	5	493	502	52	–	–	–	630	452	32
452O	4	398	382	28	–	–	–	1054	1040	22
479O	6	356	321	61	–	–	–	428	390	61
480O	7	268	365	58	–	–	–	1069	680	39

The evaluation of the participants’ results was done on the basis of a *z*-score procedure after logarithmic transformation. Thus, a *z*-score value *z*_*ik*_ for the result of measurement *i* of component *k* was determined:

1 $$ {z}_{ik}=\frac{1}{\sigma_k}{\log}_{10}\left(\frac{x_{ik}}{X_{ik}}\right) $$

*X*_*ik*_ constitutes the assigned value per dosage and *σ*_*k*_ the criterion for proficiency assessment for component *k*. The assigned value was calculated from the dosed mass concentration *c*_*ik*_ and the odor threshold *c*_0,*k*_ of the component:

2 $$ {X}_{ik}=\frac{c_{ik}}{c_{0,k}}{\mathrm{ou}}_{\mathrm{E}}/{\mathrm{m}}^3 $$

The dosed mass concentration *c*_*ik*_ was determined for each measurement based on the measurement data of the dosing device and the volume flow. The odor threshold *c*_0,*k*_ of *n*-butanol is specifically defined in EN 13725 to be *c*_0_ = 123 μg/m^3^ and the thresholds of all other components were deduced from the results reported by the participants of all proficiency tests run at HLNUG, during which the component was dosed: THT was dosed in all six proficiency tests, ETX and PIG in four tests, and RLI and AAC in two tests. Finally, the consensus value was obtained by the robust mean of the logarithmic values in accordance with standard ISO 13528 including a robust standard uncertainty for the consensus threshold value (ISO 13528 2015). Having the consensus value and standard deviation at hand subsequently allowed to calculate the consensus value and the range of uncertainty on the anti-log scale. The latter is somewhat skewed towards the upper limit so that the upper limit together with the consensus value was taken to calculate the relative standard uncertainties for the consensus threshold values of each component *u*(*c*_0, *k*_).

The criterion for proficiency assessment for each component was generally *σ*_*k*_ = 0.10, provided that Eq. (3) was met in compliance with ISO 13528, i.e., as long as the standard uncertainty *u*_*k*_ of the assigned value is small enough in comparison to the criterion for proficiency assessment.

3 $$ {\sigma}_k\ge \frac{1}{0.3}{\log}_{10}\left(1+{u}_k\right) $$

The relative uncertainty of the assigned value per component *u*_*k*_ was determined via formula (4) by considering the aforementioned robust relative standard uncertainties for the consensus threshold values of each component *u*(*c*_0, *k*_) as well as the relative uncertainty for the dosed mass concentration *u*(*c*_*k*_).

4 $$ {u}_k=\sqrt{u{\left({c}_k\right)}^2+u{\left({c}_{0,k}\right)}^2} $$

The latter was determined to be *u*(*c*_*k*_) = 1.01% for all components.

In the herein-described proficiency tests, each participant received an evaluation for all individual measurements (the abovementioned *z*-scores), as well as summary evaluations for the four different components and their whole participation. For each component, the mean value *z*_k_ of the three absolute *z*-scores per participant was calculated and taken as measure, whether the component was determined successfully (*z*_k_ < 3, result “passed”) or not (*z*_k_ ≥ 3, result “failed”). The final result was “passed,” if all four components were evaluated “passed,” otherwise the overall result was “failed”.

## Results and discussion

The results presented here rely on the outcomes of six proficiency tests performed in 2015, 2016, and 2017, analyzing six substances and mixtures (NBU, AAC, RLI, THT, PIG, which is a mixture with more than 10 different components, and ETX as a mixture of organic solvents). Thus, substances with different chemical moieties and levels of detection thresholds were chosen, some of which are potential emissions from various anthropogenic origins. For example, major sources of the volatile organic components of the ETX mixture can be industrial plants dealing with petro chemistry, surface coating, or chemical waste treatment (Schauberger et al. 2011), while the odor emissions from pigsties are infamous for being important aerial pollutants in agriculture (Hamon et al. 2012).

The evaluation of the outcomes of all six proficiency tests was done in three consecutive steps: At first, the odor threshold concentrations for all components were determined by means of a robust consensus approach. Then, the recovery rates achieved by the participants were calculated to assess the variability and the accuracy of the results. Finally, a *z*-normalization of the participant’s results permitted to objectively assess the performance of the participants and to compare them component-wise, for instance, to evaluate the suitability of *n*-butanol as reference substance in olfactometric analyses.

### Odor thresholds

Several methods exist to convert the chemical concentrations into odor concentrations (Wu et al. 2016). We resorted to use the odor threshold concentration as conversion factor between both concentration metrics. To determine the odor threshold of each component related to olfactometric normal conditions, the results of all participants were considered. In summary, 456 measurement results from 38 participants were taken into account to calculate a consensus value per component by the mean of the logarithmic values following a robust algorithm (Analytical Methods Committee 1989). Thus, the statistical procedure remains unsusceptible towards small changes in the data and is not liable to break down when facing large changes or outliers.

For *n*-butanol, an odor threshold of 106.1 μg/m^3^ (interval of extended uncertainty from 95.6 to 118.0 μg/m^3^) was calculated, which is close to the threshold defined in EN 13725 for *n-*butanol (123 μg/m^3^). For the other components, the thresholds and the respective margins are given in Table 3. Unfortunately, the uncertainty of odor threshold concentrations in the literature is rather high, probably due to differences in the applied olfactory methods and assessing panelists (Abraham et al. 2012). For example, there are evidences that the triangle odor bag method tends to deliver lower threshold concentrations than dynamic olfactometry (Wu et al. 2017). A re-determination of the thresholds is thus suggested for frequently tested odor substances to get comparable results (Wu et al. 2016).

**Table 3:** Determined odor threshold concentrations c_0_ [μg/m^3^] with lower and upper limits of extended uncertainty c_0,low_ and c_0,high_ [μg/m^3^], and calculated precision criteria *σ*_*k*_

	AAC	ETX	NBU	PIG	RLI	THT
c_0_ [μg/m^3^]	44.1	191.2	106.1	423.9	104.9	0.658
c_0,low_ [μg/m^3^]	33.0	162.7	95.6	357.5	82.5	0.576
c_0,high_ [μg/m^3^]	58.8	224.6	118.0	502.5	113.6	0.752
*σ*_*k*_	0.21	0.12	0.10	0.13	0.12	0.10

### Proficiency tests results: recoveries and variabilities

**Fig. 1:**
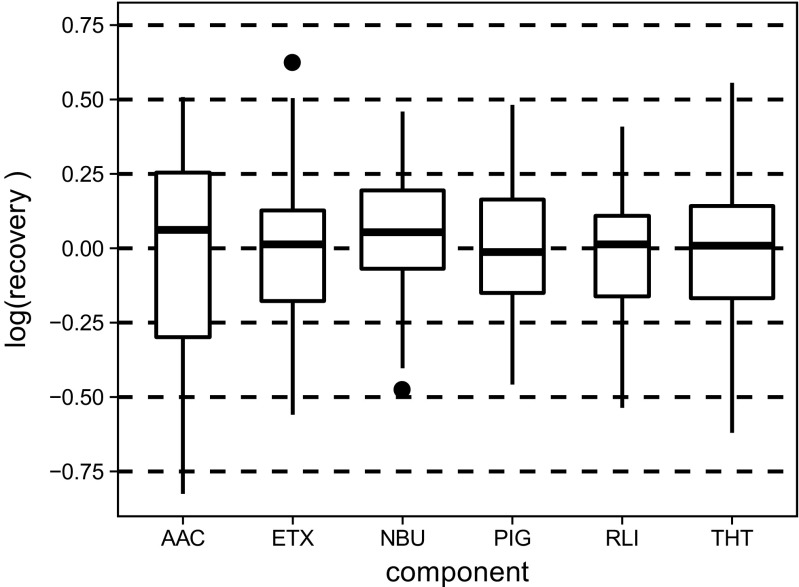
Box plot of the recoveries per component. Boxes are drawn with widths proportional to the square-roots of the number of measurements

With consensus odor thresholds at hand, an evaluation of the participants’ results was made possible. At first, mere recovery rates per component were calculated out of the single measurements to gain an insight into the degree of dispersion of the results. The boxes in Fig. 1 display the distribution of participant’s recoveries on logarithmic scale component-wise and summed up over all six proficiency tests. It is obvious that the recovery rates of AAC show the highest variability with the first and third quartile located at − 0.30 and 0.25 (i.e., recovery rates of 50 and 179%), respectively, meaning that half of the determined AAC concentrations were either lower than 50% of the true concentration or higher than almost twice the true concentration. The recovery rates of the other five components scatter on a similar scale.

In Tables 1 and 2, the assigned concentrations and the empirical mean of the measured concentrations including the relative standard deviation of the measured concentrations are provided per proficiency test. Here, comparatively large discrepancies between assigned and measured values can be found for single components (for example, NBU with 600 versus 1014 ou_E_/m^3^ (+169%) in RV430O) and mixed odorants (ETX with 516 versus 890 ou_E_/m^3^ in RV480O) as well throughout all six proficiency tests. Also, the rather large variability of the concentrations determined by the participants is striking (with relative standard deviations of up to 67% in case of AAC) and seems to be independent of the dosed concentration levels. This is not the first time that discrepancies of this magnitude were discovered. For example, three university olfactometry laboratories measured identical odor samples from agricultural swine and dairy barns (Bereznicki et al. 2012). Here, the determined odor concentrations varied as much as 50-fold in the dairy barns and 7-fold in the swine barns, whereas NBU concentrations ranged from 1.7 to 3-fold.

**Fig. 2:**
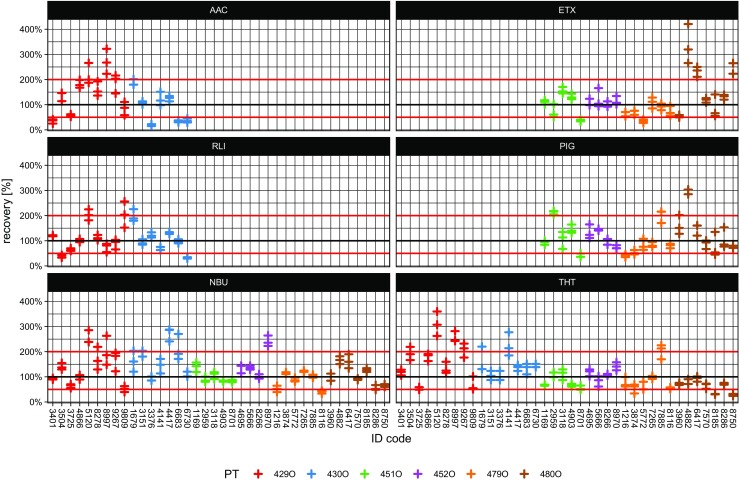
Recovery rates for each component per dosage, participant, and proficiency test (PT)

To fathom the origin of the high variabilities, the recovery rates are additionally broken down to a single-result level as can be seen in Fig. 2 for *n*-butanol and for the other components.

Even in the scope of a single proficiency test, the spread of determined concentrations of one component can be enormous: For example, during the first test in 2015 (RV429O), *n*-butanol was dosed in a concentration of 591 ou_E_/m^3^, while the measured concentrations ranged from 234 ou_E_/m^3^ (recovery of 40%) to 1682 ou_E_/m^3^ (290%). Another extreme example can be found in the ETX results obtained in 2017, where recoveries from 55 to 421% were achieved during one and the same dosing run. In certain cases, huge variations can be found even when one participant measured three almost identically concentrated samples. So, one source of variability goes down right to the smallest unit: the singular sampling and olfactometric measurement. At least for NBU and THT, a slight tendency towards tighter scatter margins from the early tests in 2015 to the latest ones in 2017 is noticeable.

### Measurement uncertainties

The observed variabilities may look overly broad and have to be compared to the expanded measurement uncertainty of olfactometric measurements of a single laboratory, which is sufficiently determined by calculating the 95% confidence interval for estimating odor concentrations. Here, the current daily standard deviation s_r_ for *n*-butanol is the major metric, for which a limit is set at s_r_ ≤ 0.1721 on a logarithmic scale by EN 13725. Thus, an extended measurement uncertainty (*k* = 2) of maximal 0.3442 or 3.44 decibel (dB) on a logarithmic scale (odor level) can be derived irrespective of the odorant. This ensures that the factor, which expresses the differences between two consecutive measurements, performed on the same testing material in one laboratory under repeatability conditions, will not be larger than 3 in 95% of the cases. The use of the standard deviation for *n*-butanol to determine the standard deviation of any number of odorant samples is based on the assumption that calculations with *n*-butanol as the reference odorant are more difficult and result in greater dispersion that is the case with actual odorant samples. So, this criterion is de facto regarded as the upper limit of the extended uncertainty for each olfactometric measurement result gained by a laboratory in compliance with EN 13725. This implies that 95% of the measurements are in the range of approximately 50 to 200% of the expected concentration. For a better illustration of the defined repeatability criterion, the red lines in Fig. 2 enclose the range of 50 to 200% of the assigned true value of *n*-butanol and the other components. As can be seen, 93 out of 114 (82%) of the results are located within the margins for *n*-butanol. The smallest within-border fraction can be found for AAC (60%) followed by PIG (74%), ETX (76%), RLI (77%), and THT (79%).

Apparently, HLNUG’s participants fared better in determining the concentration of *n*-butanol than participants from other, previously run olfactometric proficiency tests: Two conventional proficiency tests were hosted in 2003 and 2005, where only 62% of the results for *n*-butanol ended up within the span of 50 to 200% (Boeker and Haas 2007; Maxeiner 2006). In 2014, a proficiency test for dynamic olfactometry with a real odor mixture (fish sauce) was performed with the result that only 3 out of 15 participating laboratories complied with the repeatability criterion (Maxeiner 2015). These proficiency tests, however, were carried out without sampling prior to olfactometric analyses in contrast to tests presented in the underlying work.

However, the criterion can only be understood as a lower limit for uncertainty, as systematic deviations are not included and only uncertainties of the analytical and not of the sampling part are considered. For example, sampling, background odor of sampling bags, sampling storage time, and the dilution in the olfactometers might lead to additional uncertainties (Kim et al. 2012; Laor et al. 2014). To get a better estimate of the measurement uncertainty, the standard deviation of laboratory mean values (s_R_), i.e., the reproducibility standard deviation, as well as intra- (s_w_) and inter-laboratory standard deviations (s_L_) were calculated based on the logarithmized values of the odor threshold values determined by the participants. Finally, the extended measurement uncertainties U_0.95_ (*k* = 2) for each component was estimated by means of the deviation of the laboratory means s_R_. Since all data were converted by log base-10 transformation, the expanded uncertainty (*k* = 2) of a single odor measurement can be expressed as relative interval ΔU_0.95_. All calculations are based on standard ISO 5725-2 (ISO 5725-2 2002) and the results are given in Table 4.

**Table 4:** Log base-10 values of intra-laboratory standard deviation s_w_, inter-laboratory standard deviation s_L_, standard deviation of laboratory means s_R_, extended measurement uncertainty U_0.95_ and relative interval thereof ΔU_0.95_ on linear base

Odorant	NBU	AAC	ETX	PIG	RLI	THT
s_w_ [log_10_(μg/m^3^)]	0.064	0.080	0.065	0.094	0.060	0.074
s_L_ [log_10_(μg/m^3^)]	0.198	0.350	0.220	0.215	0.230	0.236
s_R_ [log_10_(μg/m^3^)]	0.208	0.359	0.229	0.235	0.238	0.247
U_0.95_ [dB]	± 4.16	± 7.18	± 4.58	± 4.70	± 4.76	± 4.94
ΔU_0.95_	38–260%	19–522%	35–287%	34–295%	33–299%	32–311%

As can be seen, each of the extended measurement uncertainties exceeds the criterion given in the standard EN 13725 (3.44 dB). This is no surprise, because the underlying reproducibility precision s_R_ incorporates not only uncertainty portions specific for single laboratories, but also different variations between laboratories, due to, e.g., selection strategies of assessors by olfactometer operators, presentation schemes of dilutions to assessors, or calibration procedures. Thus, s_R_ is a valid estimator for the uncertainty of a measurement procedure in contrast to the usually applied current daily standard deviation s_r_ for *n*-butanol. For all components but AAC (ca. 20–520%), the extended range of uncertainty around a single olfactometric measurement value goes approximately from 33 to 300%. For NBU, a reproducibility standard deviation of s_R_ = 0.208 implies that the difference between two single measurements, performed on testing material originating from one source in two or more laboratories under reproducibility conditions, will be no larger than factor 4 in 95% of cases. For the other components, factors from 4.4 (ETX) to 10 (AAC) were determined. Interestingly, the within-laboratory standard deviations s_w_ clearly meet the criterion of the standard EN 13725, namely s_r_ ≤ 0.1721, although s_w_ considers additional uncertainties due to the sampling method.

### Proficiency test results: *z*-scores

**Fig. 3:**
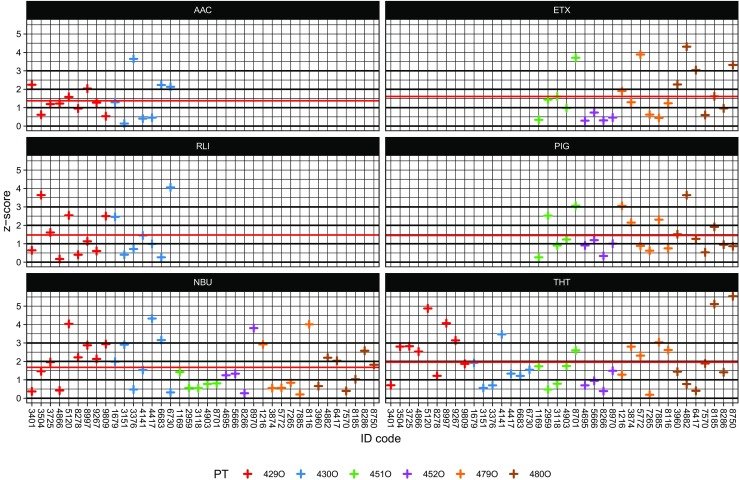
Mean *z*-scores per component, participant, and proficiency test (PT); the red lines depict the mean of *z*-scores per component

To harmonize the participants’ outcomes from different components and proficiency tests, the recovery results are converted into *z*-scores after logarithmic transformation following formula (1). Log-transformation prior to calculating *z*-scores is effective in establishing near-symmetric distributions that are sufficiently close to normal to justify interpretation on the basis of the normal distribution (Thompson et al. 2006). Usually, *z*-scores from a typical round of proficiency test resemble a standardized normal variate so that participants with a persistent analytical bias or a large run-to-run standard deviation would produce an undue proportion of *z*-scores outside a predefined limit. The occurrence of out-of-bound scores (scores outside the range of ± 3) is usually taken as requiring investigation or even remedial actions (Analytical Methods Committee No 68 2016). Finally, a *z*-score would be valid as guide for action only if the criterion for proficiency assessment has an uncertainty that is fit for purpose (Analytical Methods Committee No 74 2015). Otherwise, unsatisfactory scores might be caused due to inaccuracies in the determination of the assigned value and the assigned threshold and not because of lacking competence of the participant. Thus, the criterion for proficiency assessment for each component *σ*_*k*_ has been adapted according to Eq. (3) and is given in Table 3. In the end, three *z*-scores per component and participant were calculated and the mean of the absolute values was taken as measure, whether the component was determined successfully (*z*_*k*_ < 3) or not. Figure 3 provides an overview of the final *z*-scores per participant.

As the calculated *z*-scores are based on a logarithmic scale, the *z*-scores stand for rather large margins on the linear scale, e.g., for a given criterion for proficiency assessment of *σ*_*k*_ = 0.10, deviations from the true value in the range of 50 to 200% still deliver results of *z*_k_ < 3. Twenty-three out of 152 results exceeded this criterion, whereas 102 brought forth satisfactory results with *z*_k_ < 2. All in all, the results for the mixtures (average *z*-score 1.61 for ETX, 1.45 for PIG) are comparable to those of single odorants (1.68 for NBU, 1.47 for RLI), whereas AAC (1.37) and THT (1.96) showed the lowest and highest average *z*-scores, respectively. The participants passed the whole proficiency test in case all of their component-wise *z*-scores are below 3. Thus, 50% (19 out of 38) of the participants would have passed the proficiency test. It shall be taken into account that the results of this retrospective analysis are based on the cumulated data of all six proficiency tests, whereas each test was evaluated individually in the year it was hosted. Especially, the determined odor threshold values and precision criteria make all the difference, except for *n*-butanol. Thus, the results presented here do not necessarily concur with the outcomes of the single proficiency tests.

### *n*-Butanol: a truly reliable olfactometric reference?

**Fig. 4:**
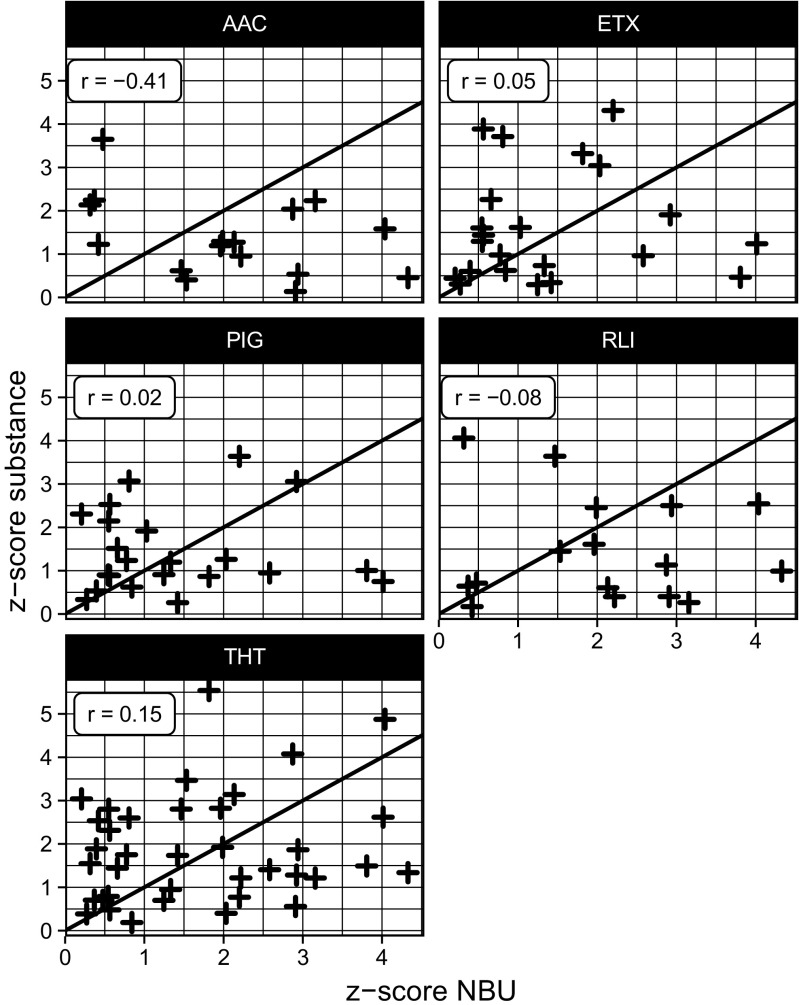
Comparison of the *z*-scores of the other components with *n*-butanol together with angle bisector (black line), and correlation coefficient r

Being the reference material for olfactometry in accordance with EN 13725, it is worthwhile to compare the performances regarding *n*-butanol to the other components. *n*-Butanol functions as reference as long as two premises are given: traceability (equivalence between 1 ou_E_ *n*-butanol and 1 ou_E_ of any other odorant) and predictability so that a panelist’s sensitivity to *n*-butanol is regarded as measure for the panelist’s sensitivity to other odorants. If the latter was given, then a correlation between the *n*-butanol results and the results for the other odorants would be recognizable. To test this assumption, a comparison was made between the *z*-scores of *n*-butanol and the other components in Fig. 4. If the participant determined the respective component as (un)successful as *n*-butanol, a point directly on the angle bisector (black line) would be the result. Points below the angle bisector thus stand for a better performance in quantifying the very component in regard to *n*-butanol and vice versa.

For none of the components, an obvious correlation is apparent. So it is not surprising that also the correlation coefficients between the scores of *n*-butanol and the other components are not significant (r = − 0.41 for AAC to 0.15 for THT). One reason might be given by a publication by Zernecke et al., in which it is assumed that only odorants with corresponding functional groups might activate the same olfactory receptors within the panelists so that only in this case a performance transfer is valid (Zernecke et al. 2011). This seems logical, because even the stereochemistry of otherwise chemically identical molecules heavily influences threshold concentrations (Gallagher et al. 2015). Furthermore, Klarenbeek et al. analyzed outcomes of 10 proficiency tests comprising *n*-butanol and other odors and found out that important components of variance significantly differed between *n*-butanol and other odorants (Klarenbeek et al. 2014). In another publication, a predictability of panelists’ sensitivity to pig house odor based on their sensitivity to *n*-butanol could not be confirmed (McGinley and McGinley 2006). This again arises the question whether *n*-butanol as reference substance is suitable in the process of panelist selection and whether other single- or multi-component references better reflect the chemical makeup of odorant emissions into air. By using empirically determined odor thresholds, effects of mixing of multiple odorants like masking or dominance by stronger odorants, so that the odorous substances do not behave additively, can be neglected (Kim and Kim 2014; Thomas-Danguin et al. 2014). Considering in this respect the results in the present study, there seems to be no contradiction to use other substances than *n*-butanol as reference material and for panel selection purposes. Also, the usage of odorous mixtures like ETX and PIG did not prove advantageous in this respect. It still remains inconclusive, whether more complex chemical stimuli due to a mixture of multiple odors can provide more reliable results than single-type odorants (Laska and Hudson 1991; Oleszkiewicz et al. 2017). However, it is safe to assume that a selection of reference compounds should contain most of the functional groups, which are usually encountered in gases of interest and which are responsible for activating the assessor’s olfactory receptors.

## Conclusion

Dynamic olfactometry is the most commonly applied method in Europe to assess odor emissions from different sources and to evaluate the efficiency of odor reduction techniques. To do so, a well-defined procedure to describe the odor strength is imperative, which is why the standard EN 13725 was published in 2003. Over the years, however, doubts were raised by users and researchers concerning discrepancies between the standard’s performance criteria on one hand and measurement uncertainties achieved when olfactometry is conducted in reality on the other hand. In this respect, also the transferability of performance characteristics from *n*-butanol to other odorants and mixtures is much-debated. In order to formulate clear answers to these questions, a study under proficiency test conditions was conducted.

In the course of six olfactometric stack emission proficiency tests, highly variable recovery rates of all the analyzed odorants were discovered. The sampling process itself seemed to be of minor importance, as the spread of results is comparable to outcomes of former, purely analytical proficiency tests without sampling. The derived extended measurement uncertainties range from circa 30 to 300% for *n*-butanol and all the other tested substances apart from amyl acetate with a larger range (20–520%). The reproducibility standard deviation for *n*-butanol was 0.208 meaning that in 95% of cases, the difference between two single measurements performed on the same testing material by two or more laboratories under reproducibility conditions can be as large as factor 4. For the other analyzed odorants, factors of 4 to 10 were derived. These outcomes are in stark contrast to the standard EN 13725, which allows to calculate the 95% confidence interval for estimating the expected odor concentration with the aid of the laboratory’s current daily standard deviation for *n*-butanol bringing forth a critical quality range of only 45–220% and allowing a maximal factor between two single measurements under reproducibility conditions of only 3.

None of the analyzed odor components proved significantly better or worse than the others independent of the fact that single- and multi-component odorous agents were analyzed. Furthermore, an objective comparison of *z*-normalized results between the analytes did not give an empirical proof as to why *n*-butanol or odorous mixtures should be more qualified as reference olfactometric substance than other single-agent odors. Or in other words: The high variability of the olfactometric measurement data is an inherent problem of the (analytical) method itself and not of the fact that *n*-butanol is an unsuitable reference material. Whether data scattering in the present magnitude is acceptable in legally regulated areas remains to be discussed among experts.

Another weak point of olfactometry is the high uncertainty of odor threshold concentrations in the literature, which are an important prerequisite to convert mass concentrations to odor concentrations and vice versa. Thus, for each odorant, an odor threshold was determined based on a robust consensus approach and was utilized to determine the assigned concentrations per dosage. Doing so was imperative as either published thresholds vary considerably or are not known at all, and underlines the need for a collaborative endeavor to determine approved thresholds for frequently analyzed odorants. The odor threshold concentrations presented in this publication should be quite reliable for measurements conforming to EN 13725, as they represent robust means of independent measurements performed by 38 accredited laboratories.
